# Dasatinib-Related Chylothorax

**DOI:** 10.4274/tjh.2012.0196

**Published:** 2015-02-15

**Authors:** Yen-Min Huang, Cheng-Hsu Wang, Jen-Seng Huang, Kun-Yun Yeh, Chien-Hong Lai, Tsung-Han Wu, Pei-Hung Chang, Yueh-Shih Chang, Yii-Jenq Lan

**Affiliations:** 1 Chang Gung Memorial Hospital, Clinic of Internal Medicine, Division of Hematology-Oncology, Keelung, Taiwan; 2 Chang Gung Memorial Hospital, Clinic of Internal Medicine, Division of Hematology, Linkou, Taiwan; 3 Chang Gung University Faculty of Medicine, School of Traditional Chinese Medicine, Taoyuan, Taiwan

**Keywords:** Dasatinib, Chylothorax, Chronic myeloid leukemia

## Abstract

Dasatinib is a potent second-generation tyrosine kinase inhibitor for the treatment of chronic myeloid leukemia. The most common adverse event associated with dasatinib therapy is fluid retention, including pleural effusion. Dasatinib-related chylothorax has rarely been reported. The clinical manifestations, pathophysiology, management, and prognosis are not fully understood. Here we report a 40-year-old woman presenting with chylothorax following dasatinib use. We propose the hypothesis of its mechanism as well as offering a review of the relevant literature.

## Abstract (tr)

Dasatinib kronik miyeloid lösemi tedavisinde kullanılan etkin bir ikinci kuşak tirozin kinaz inhibitörüdür. Dasatinib tedavisi ile ilişkili en sık görülen yan etki plevral effüzyonu da içeren sıvı retansiyonudur. Dasatinib ile ilişkili şilotoraks nadiren bildirilmiştir. Klinik belirtileri, patofizyolojisi, yönetimi ve prognozu tam olarak anlaşılamamıştır. Burada dasatinib kullanımı sonrasında şilotoraks gelişimi ile başvuran 40 yaşında bir kadın sunulmuştur. Dasatinib ile ilişkili şilotoraks gelişiminin mekanizmasının yanı sıra, konu ile ilgili literatür derlemesinden de bahsedilmektedir.

## INTRODUCTION

Dasatinib (Sprycel®) is a highly potent small-molecule inhibitor of BCR-ABL and Src family tyrosine kinases, which is indicated for the treatment of adults with newly diagnosed chronic-phase chronic myeloid leukemia (CML), resistance or intolerance to prior CML therapy, or Philadelphia chromosome-positive (Ph+) acute lymphoblastic leukemia (ALL). Pleural effusion was a common complication in 14%-32% of all patients in clinical trials [1,2,3]. However, cases of dasatinib-related chylothorax have rarely been reported. The pathophysiology and management of chylothorax remain unclear. It is essential to provide more information about dasatinib-related chylothorax since dasatinib is increasingly being used due to its effectiveness in the treatment of CML and Ph+ ALL.

## CASE PRESENTATION

A 40-year-old female, a housewife, had been diagnosed with CML with b2a2 BCR-ABL fusion 8 years ago. The initial presentation was peripheral leukocytosis with splenomegaly. She received 400 mg imatinib daily after the diagnosis and was titrated to 300 mg twice a day due to slow log reduction of BCR-ABL after 2 years. The log reduction achieved major molecular response half a year later. Four years after the diagnosis, the treatment was shifted to 50 mg of dasatinib twice a day because of intolerable knee pain. Her knee pain improved soon and the log reduction achieved complete molecular response (CMR) after 3 years of dasatinib treatment. Her liver and renal functions were monitored regularly and were within normal ranges.

**Figure 1:**
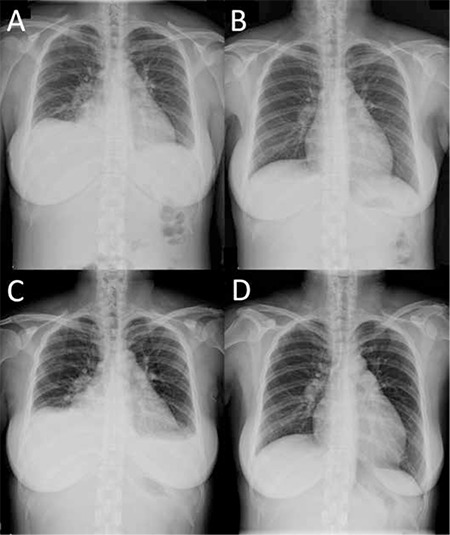
Chest radiography: A) initial small pleural effusion; B) 9 days later after diuretics and steroid treatment; C) recurrent pleural effusion after dasatinib was resumed; D) 6 weeks after dasatinib interruption.

Forty months after dasatinib use, the patient complained of progressive dyspnea and aggressive cough. Chest radiography revealed elevation of the right hemidiaphragm and small pleural effusion of the left (Figure 1A). Computed tomography (CT) confirmed prominent right pleural effusion and a small amount over the left side, as well as pericardial effusion. Echocardiography revealed adequate left ventricular function. Liver cirrhosis and splenomegaly were excluded by abdominal sonography. Ascites formation was excluded by the above CT and sonographic studies. Chest ultrasound-guided thoracentesis of bilateral pleural fluid yielded exudative effusion to be compared with her serum total protein concentration (6.4 g/dL) and lactic dehydrogenase (LDH) concentration (244 U/L) (Table 1) according to Light’s criteria [4]. Bilateral chylothorax was determined based on the milky yellow appearance and elevated triglyceride concentration (right: 263 mg/dL, left: 536 mg/dL) of the pleural effusion. Her serum triglyceride was 80 mg/dL, similar to her previous levels. Culture was negative for bacteria and tuberculosis. Cytology of the pleural effusion showed lymphocytes and macrophage/mesothelial cells. The patient denied history of trauma or surgery. The etiologies of chylothorax including trauma, surgery, infection, or malignancy were not likely. Under the suspicion of drug-related chylothorax, dasatinib was discontinued. Diuretics and steroids were prescribed for symptom control. Her pleural effusion improved after 9 days of treatment (Figure 1B). Dasatinib was resumed 2 weeks after discontinuation and the pleural effusion recurred soon under the treatment of diuretics and steroids (Figure 1C). Due to intractable chylothorax even after repeated thoracentesis, dasatinib was discontinued again and the patient was later switched to nilotinib. The pleural effusion resolved gradually (Figure 1D). After several months of nilotinib use, no further symptoms of pleural effusion were experienced. Informed consent was obtained.

## DISCUSSION AND REVIEW OF THE LITERATURE

Dasatinib (Sprycel®) is a second-generation BCR-ABL tyrosine kinase inhibitor that targets most imatinib-resistant BCR-ABL mutations (except the T315I and F317V mutants) by distinctly binding to active and inactive ABL-kinase [5]. It was first used as second-line treatment for imatinib-resistant or -intolerant patients with CML [6]. Later, dasatinib was considered as first-line therapy for patients with CML, and further clinical trials are presently continuing [1]. Common adverse effects of dasatinib include body fluid retention (all grades, up to 35%), skin rash (all grades, 10% to 20%), and diarrhea (all grades, 3% to 31%) [7]. Among body fluid retention, pleural effusion was seen in 14%-32% of all patients in clinical trials of dasatinib [1,2,3]. The pathophysiology of dasatinib-related chylothorax is still not fully understood.

Chylothorax is caused by chyle leakage from the thoracic duct into the pleural space, which results from disruption or obstruction of the thoracic duct. Chyle typically contains high levels of triglycerides and often has a turbid and milky appearance. Triglyceride concentrations of pleural effusion greater than 110 mg/dL (1.24 mmol/L) strongly support the diagnosis [8]. In this case, the patient’s triglyceride concentration levels were 536 mg/dL in the left pleural space and 263 mg/dL in the right. Those findings supported the diagnosis of bilateral chylothorax. The most common etiology of chylothorax is surgery or trauma [9], accounting for nearly half of all cases. Nontraumatic etiologies include malignancy, sarcoidosis, retrosternal goiter, amyloidosis, superior vena cava thrombosis, benign tumors, congenital duct abnormalities, and diseases of the lymph vessels such as yellow nail syndrome, lymphangioleiomyomatosis, and hemangiomatosis [10]. Hematologic malignancies including lymphoma, chronic lymphocytic leukemia, and Waldenström macroglobulinemia were reported to be associated with chylothorax [11,12,13,14,15]. Among the above hematologic malignancies, non-Hodgkin’s lymphoma is the most common. Only one case report mentioned CML and chylothorax, which was Adams-Oliver syndrome-related [16]. There is no current evidence of the association between the natural course of CML and chylothorax. In our case, the patient’s pleural effusion was regarded as concordant exudate according to an analysis of chylothorax [17]. The causes of this kind of chylothorax include lymphoma, tuberculosis, empyema, and idiopathic/biliopleural fistula. Infection was not likely as the culture results for tuberculosis and bacteria were negative. There was no evidence of lymphoma. It is most likely that the cause of the chylothorax was dasatinib therapy.

Dasatinib-related pleural effusions are generally lymphocyte-predominant exudates [18]. These findings suggest that the etiology of pleural effusions is different from that of body fluid retention. The exact mechanism of pleural effusions remains unclear. The possible mechanisms potentially include inhibiting platelet-derived growth factor receptor beta (PDGFR-β) expressed in pericytes, which is involved in the regulation of angiogenesis [19]. The defect of PDGFR-β is associated with the formation of abnormal initial lymphatics in human lymphedema distichiasis, whereas another report suggested that platelet-derived growth factor (PDGF)-BB and its receptor, PDGFR-β, are directly lymphangiogenic [20,21]. This mechanism was reported in Gorham’s disease-related chylothorax [22].

Src kinase inhibition by dasatinib is also possibly related to changes of vascular endothelial growth factor-mediated vascular permeability and stability of the pleural epithelium [23,24]. The PDGF-signaling pathway stimulates tumor cell proliferation, angiogenesis, and pericyte recruitment to tumor blood vessels. Furthermore, ligated integrins recruit several nonreceptor tyrosine kinases, including focal adhesion kinase, integrin-linked kinase, and Src-family kinases, among others [25]. In congenital chylothorax of human fetuses, human integrin defect genes were found [26]. If the presentation of Src kinases changes, it may lead to defects of integrin and further chylothorax.

The management of dasatinib-related pleural effusion including diuretics and a short course of prednisone (40 mg daily for 4 days) was suggested [18]. Once-daily dosing would reduce incidence of pleural effusions versus twice-daily dosing. There is no established standard treatment for chylothorax. In an earlier report, 138 patients were treated with dasatinib as second-line treatment after imatinib failure [27]. Forty-eight patients had pleural effusion and 1 of them developed recurrent chylous effusions, which required 12 treatments of thoracentesis. In another report, 40 patients were treated with dasatinib and 6 of them had pleural effusion [28]. One patient had right-sided chylothorax after about 1 year of dasatinib treatment and improved after drug interruption. In our case, the patient was treated with thoracentesis, prednisolone, and diuretics first, but the pleural effusions recurred soon after dasatinib was resumed. The second disappearance of chylothorax after discontinuing dasatinib again suggests the association between dasatinib and chylothorax. We therefore changed her treatment to another tyrosine kinase inhibitor, nilotinib. Now she maintains her CMR status.

In conclusion, in patients under dasatinib treatment who develop chylothorax, dasatinib-related chylothorax should be considered. Dasatinib should be discontinued if the chylothorax is intractable. Further investigation to define the pathogenesis of dasatinib-related chylothorax is warranted since dasatinib is increasingly being used.

## Figures and Tables

**Table 1:**
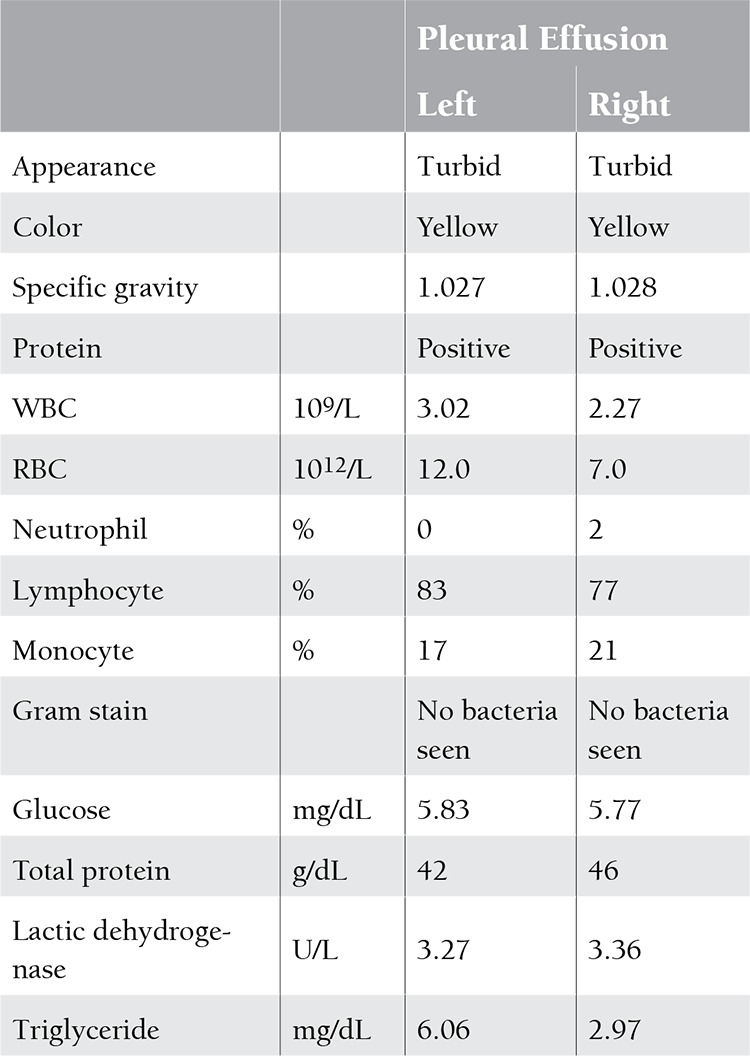
Pleural effusion studies.
